# Deep learning-based multimodal image analysis predicts bone cement leakage during percutaneous kyphoplasty: protocol for model development, and validation by prospective and external datasets

**DOI:** 10.3389/fmed.2024.1479187

**Published:** 2024-09-19

**Authors:** Yu Xi, Ruiyuan Chen, Tianyi Wang, Lei Zang, Shuncheng Jiao, Tianlang Xie, Qichao Wu, Aobo Wang, Ning Fan, Shuo Yuan, Peng Du

**Affiliations:** ^1^Department of Orthopedics, Beijing Chaoyang Hospital, Capital Medical University, Beijing, China; ^2^Department of Spine Surgery, Beijing Shunyi Hospital, Beijing, China

**Keywords:** osteoporotic vertebral compression fracture, percutaneous kyphoplasty, bone cement leakage, artificial intelligence, deep learning

## Abstract

**Background:**

Bone cement leakage (BCL) is one of the most prevalent complications of percutaneous kyphoplasty (PKP) for treating osteoporotic vertebral compression fracture (OVCF), which may result in severe secondary complications and poor outcomes. Previous studies employed several traditional machine learning (ML) models to predict BCL preoperatively, but effective and intelligent methods to bridge the distance between current models and real-life clinical applications remain lacking.

**Methods:**

We will develop a deep learning (DL)-based prediction model that directly analyzes preoperative computed tomography (CT) and magnetic resonance imaging (MRI) of patients with OVCF to accurately predict BCL occurrence and classification during PKP. This retrospective study includes a retrospective internal dataset for DL model training and validation, a prospective internal dataset, and a cross-center external dataset for model testing. We will evaluate not only model’s predictive performance, but also its reliability by calculating its consistency with reference standards and comparing it with that of clinician prediction.

**Discussion:**

The model holds an imperative clinical significance. Clinicians can formulate more targeted treatment strategies to minimize the incidence of BCL, thereby improving clinical outcomes by preoperatively identifying patients at high risk for each BCL subtype. In particular, the model holds great potential to be extended and applied in remote areas where medical resources are relatively scarce so that more patients can benefit from quality perioperative evaluation and management strategies. Moreover, the model will efficiently promote information sharing and decision-making between clinicians and patients, thereby increasing the overall quality of healthcare services.

## 1 Introduction

The incidence of osteoporosis is escalating as the global population ages, causing a concomitant increase in osteoporotic vertebral compression fracture (OVCF). Annually, approximately 1.4 million new onset OVCFs are reported globally (1–3). OVCF causes low back pain, vertebral deformity, and severe functional impairment, which profoundly diminish the quality of life in the elderly population and impose substantial burdens on families and healthcare systems (4–6). Fortunately, percutaneous kyphoplasty (PKP) has become a widely adopted and effective clinical intervention for OVCF. PKP, as a minimally invasive approach, exhibits several merits, including substantial pain alleviation, partial vertebral height restoration, and early mobilization facilitation (7, 8).

However, bone cement leakage (BCL) has drawn significant attention as one of the most prevalent PKP complications, with incidence rates ranging of 5–80% (9, 10). Although a large proportion of BCLs are asymptomatic, poor outcomes still occur in specific individuals. Meanwhile, the type of complications significantly varies among different leakage locations. In particular, cement leakage into the spinal canal causes spinal cord and/or nerve root compression, potentially causing radicular pain and neurological dysfunction, which may require additional nerve decompression surgery (11). Furthermore, cement leakage into the paravertebral vein may cause serious conditions such as pulmonary embolism, cardiac perforation, cerebral embolism, and even fatality (12, 13). Additionally, cement leakage into the intervertebral disk exacerbates disk degeneration and increases the risk of adjacent vertebrae fractures (11, 14). Consequently, minimizing the incidence of BCL has become a crucial concern for surgeons. However, effective and intelligent methods to precisely predict BCL preoperatively remain lacking.

In recent years, the advent of deep learning (DL) has greatly facilitated the clinical applications of predictive models and the development of precision medicine. DL directly uses medical images as input, thereby automatically extracting effective features and leveraging computational power to integrate multimodal medical data, to construct more complex and high-performance predictive models, compared with traditional machine learning (ML) (15). However, previous studies predominantly used traditional ML techniques, mostly the traditional logistic regression models, to predict BCL (16–18). Such models somehow help clinicians in predicting BCL, but several limitations remain. First, manual imaging feature extractions are predominantly applied, which is not only time-consuming but also introduces subjectivity and selectivity, thereby potentially omitting impactful features. Secondly, no further BCL classification was conducted by current predictive models, limiting their clinical application. To the best of our knowledge, no study has developed a DL model to directly predict BCL during PKP based on preoperative medical images, which is quite difficult but valuable.

Therefore, this study aims to utilize multimodal analysis of preoperative computed tomography (CT) and magnetic resonance imaging (MRI) of patients with OVCF to develop a DL-based prediction model for accurately predicting BCL occurrence and classification during PKP. Moreover, the study will test the generalization ability of the model in prospective and multicenter datasets. The model will help spine surgeons accurately and conveniently determine patients at high risk for BCL and provide further personalized treatment options.

## 2 Methods

### 2.1 Study design

**Figure 1:**
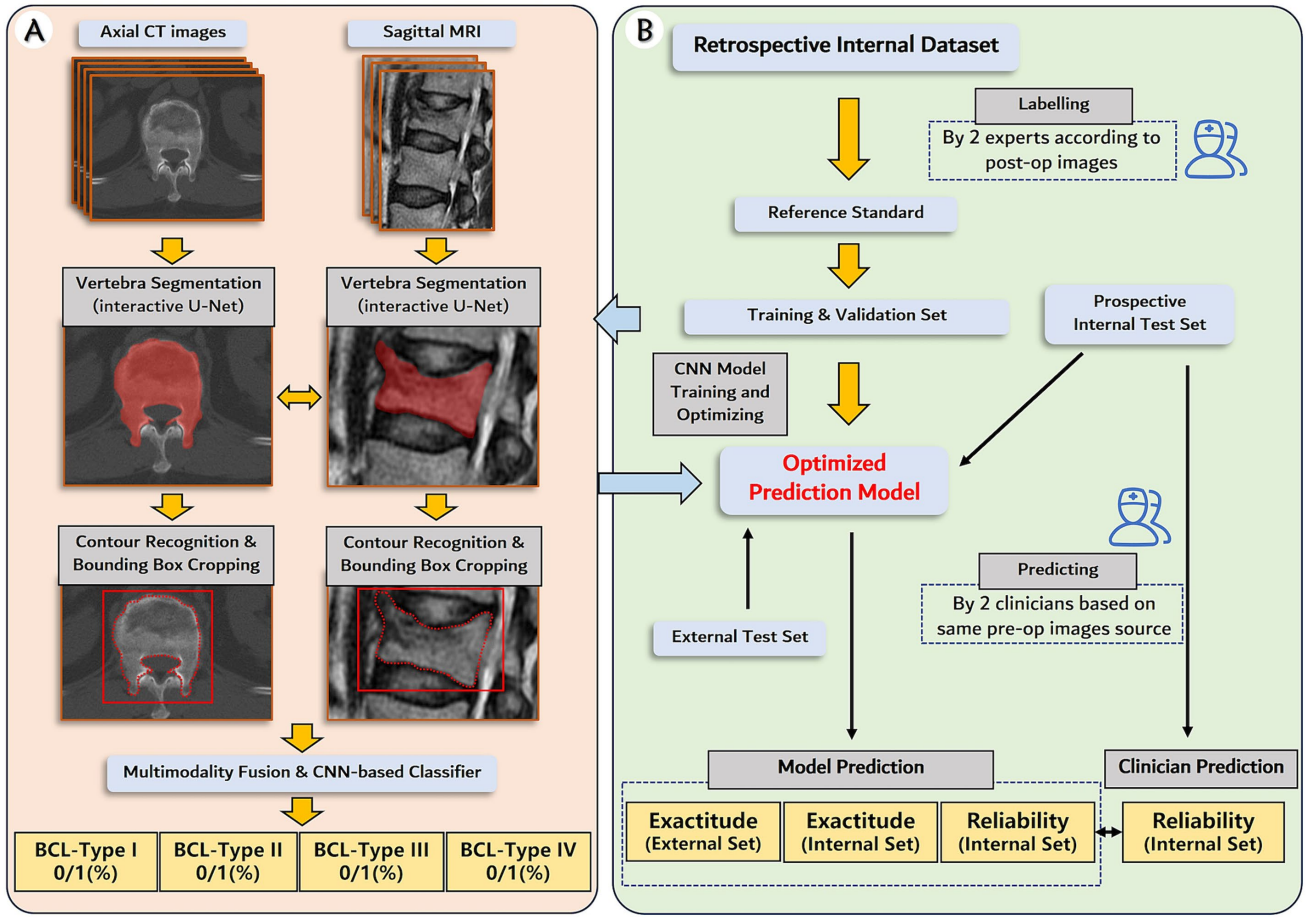
Establishment and validation of deep learning prediction model for the subtypes of bone cement leakage. **(A)** The pipeline of the deep learning model. The model utilizes matched preoperative axial CT images and sagittal MRIs as input, followed by vertebra segmentation and contour recognition to generate a bounding box. The bounding box contains but is not limited to the whole vertebra volume. Next, intermediate multimodal fusion CNN-based classifiers will be used to integrate the information of the different modalities, and complete the multitask outputs. **(B)** Flowchart of the study design, including datasets, labeling, model development, and performance evaluation.

This study will develop a DL-based prediction system for preoperatively predicting the four subtypes of BCL during PKP based on a retrospective internal dataset, and test its performance via prospective internal dataset and cross-center external dataset (Figure 1B). Approval for the protocol has been obtained from the ethics committees of our institution. Informed consent to this study is waived as its retrospective collection of preoperative data. This study protocol was conducted following the Transparent Reporting of a multivariable prediction model for Individual Prognosis or Diagnosis (TRIPOD) statement for developing the prediction model (Supplementary Table 1) (19). Our reporting will be updated if the TRIPOD-AI statement is published during our research (20).

### 2.2 Population

This retrospective study includes a retrospective internal dataset for DL model training and validation, a prospective internal dataset, and a cross-center external dataset for model testing. A retrospective internal dataset will be collected from consecutive inpatients of Beijing Chaoyang Hospital from January 2016 to July 2023. A prospective internal test dataset will be drawn from a prospective cohort established from August 2023 to March 2024 in Beijing Chaoyang Hospital for evaluating postoperative complications, consisting of consecutive patients undergoing PKP for treating OVCF. This prospective cohort contained intact perioperative data of participants and was registered on the Chinese Clinical Trial Registry (ChiCTR2300073507). The external dataset will be retrospectively collected from consecutive inpatients of Beijing Shunyi Hospital from January 2022 to January 2024.

### 2.3 Inclusion and exclusion criteria

Inclusion and exclusion criteria are identical for both internal and external datasets. Inclusion criteria include: (1) age > 55 years; (2) diagnosis of acute or subacute OVCFs; (3) complete preoperative image data within 2 weeks preoperatively, including spine X-ray, CT, and MRI; and (4) undergoing PKP for treating OVCF. The diagnosis of acute or subacute OVCF was based on a comprehensive evaluation of history, physical examination, and radiographic evidence, which is elaborated as follows: (1) meeting the World Health Organization diagnostic criteria for osteoporosis (21), and (2) severe back pain occurred within 3 weeks, aligning with (3) tenderness or percussion pain at corresponding vertebral level, and (4) a decrease in vertebral body height or cortical disruption showed on spine X-ray, CT, or MRI, and (5) bone marrow edema shown on fat suppression T2-weighted images of MRI.

Exclusion criteria include: (1) reoperation on the index vertebral level previously undergone PVP or PKP; (2) spinal tumor, infection, or severe deformity; (3) incomplete preoperative demographic or clinical baseline data; (4) patients with neither immediate postoperative spine CT nor X-ray (within 1 week postoperatively); and (5) low image quality of spine CT or MRI.

### 2.4 Surgical technique

Percutaneous kyphoplasty was performed in the prone position under local anesthesia, and all procedures were conducted using a unilateral or bilateral transpedicular approach. A needle and an inflatable balloon were inserted through the working channel into the fractured vertebral body under visualization with lateral and anteroposterior fluoroscopy. A kyphoplasty balloon was then used to inflate and create a cavity. Subsequently, the balloon was deflated and removed, followed by filling with viscous polymethylmethacrylate. The cannula was removed after the bone cement was fixed. Patients were allowed to move around gradually under the protection of thoracolumbosacral orthosis 2–3 days postoperatively.

### 2.5 Date collection

First, preoperative demographic and clinical baseline data of eligible participants, including age, sex, body mass index (BMI), bone mineral density (BMD), time from injury to surgery, fracture location, and previous OVCF, will be collected according to the medical records.

Besides, this study will collect matched preoperative spine CT images and MRIs of each patient for DL model training and validation. For both CT and MRI scan, all subjects are scanned in supine position. Image acquisition details of the internal dataset are described as follows: CT data acquisition of the spine are performed on a 64-slice MDCT scanner (Discovery CT750 HD, GE Healthcare, United States). A slice thickness of 1.25 mm and an in-plane resolution of 512 × 512 pixels were set for CT scans. Spine MRI is performed using a 3.0-T magnetic resonance system (Magnetom Verio, Siemens, GER). The scan parameters are as follows: T1WI (TR/TE, 400–600/8 ms), T2WI (TR/TE, 3,000–3,100/100 ms), T2WI-FS sequence (TR/TE, 4,000–4,200/70–80 ms), axial in-plane resolution: 256 × 224 pixels, axial slice thickness 3 mm, and intersection gap of 0.5 mm. Sagittal images were then reconstructed with an in-plane resolution of 320 × 256 pixels and a slice thickness of 4 mm. Noteworthily, most patients with OVCF usually complain of back pain at first visit, which sometimes is difficult to differentially diagnosed with degenerative spinal disease, especially in whom without definite trauma history. Hence, the reserved initial MRI may not contain continuous axial images at the fracture vertebral level in specific individuals. Therefore, we choose to collect continuous sagittal T2WI of MRI combined with continuous axial CT images, to obtain maximum information from multimodal image sources. For external dataset, spine CT is performed on a 16-slice CT scanner (SOMATOM Emotion 16, Siemens Healthcare, Germany). A slice thickness of 3.75 mm and an in-plane resolution of 512 × 512 pixels were set. MRI is performed using the same 3.0-T magnetic resonance system (Magnetom Verio, Siemens, GER) as in the internal dataset. The scan parameters were generally same except for an axial in-plane resolution of 384 × 269 pixels, an intersection gap of 0.3 mm, and a sagittal in-plane resolution of 384 × 326 pixels. All image data will be stored in DICOM files for further processing. The retrospective internal dataset will be divided into training and validation sets at a 9:1 ratio.

The procedure of data collection will be conducted by two junior spine clinicians (RC with 2 years of experience and YX with 2 years of experience) for the internal dataset and one junior spine clinician (TX with 3 years of experience) for the external dataset. These clinicians are not in charge of all involved patients and are blinded to the group allocation and outcome assessment.

### 2.6 Diagnostic criteria for BCL and dataset labeling

**Figure 2:**
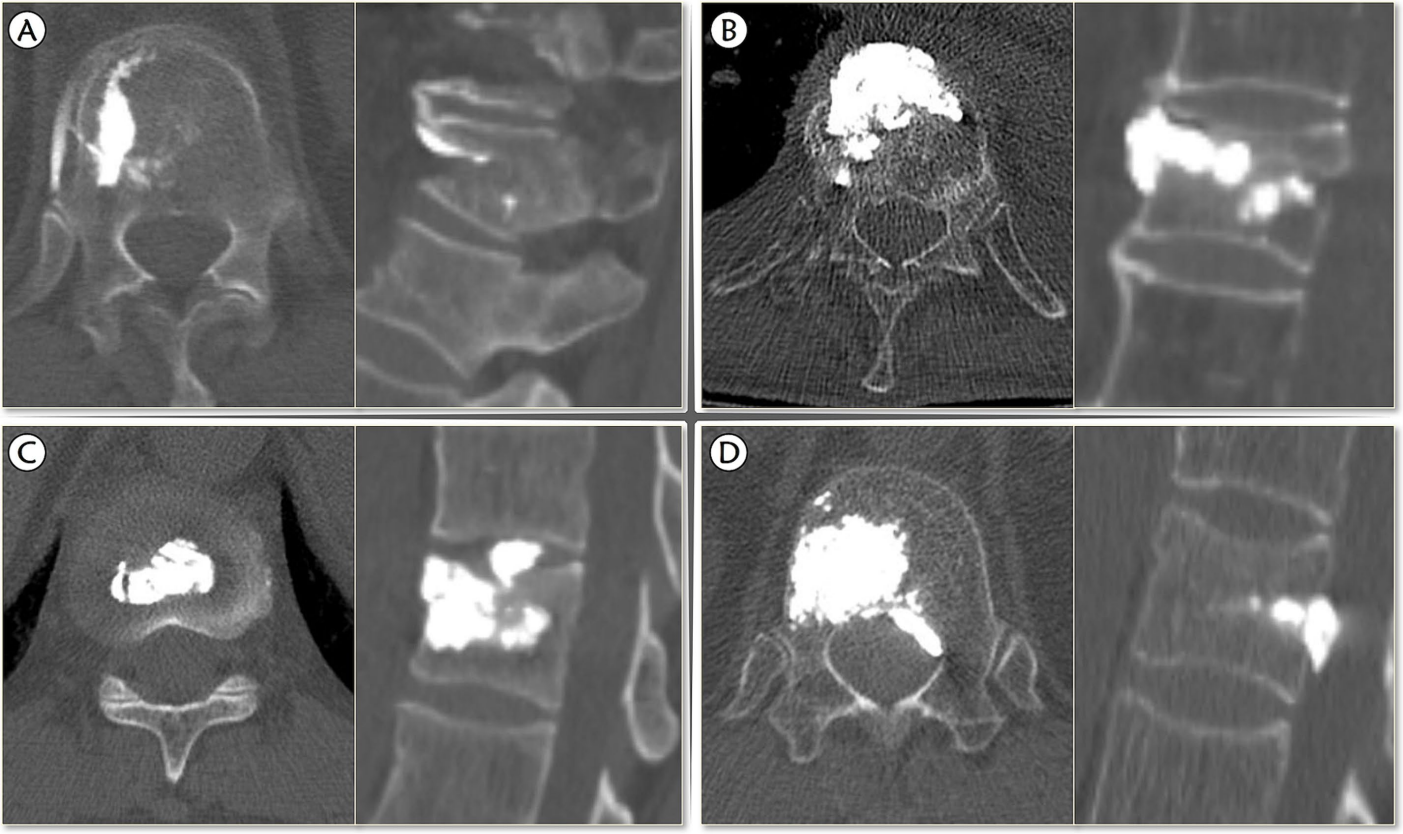
The modified classification of bone cement leakage. **(A)** Type I, leaking through the segmental vein; **(B)** Type II, leaking through the cortical defect to perivertebral soft tissue; **(C)** Type III, leaking into intervertebral disk; **(D)** Type IV, leaking into the spinal canal (through basivertebral vein or cortical defect).

Although postoperative CT is the most accurate approach to detect BCL, it is not necessary for all types of BCL (22), and the postoperative X-ray can achieve satisfactory sensitivity (23). Therefore, either the immediate postoperative spine CT or X-ray will be checked to confirm the occurrence of BCL. BCL was defined as the presence of extravertebral cement. Further, we proposed modified four subtypes of BCL are differentiated by the location of the extravertebral cement according to Bermejo et al. (9): (1) Type I, leaking through the segmental vein; (2) Type II, leaking through the cortical defect to perivertebral soft tissue; (3) Type III, leaking into intervertebral disk; (4) Type IV, leaking into the spinal canal (through basivertebral vein or cortical defect) (Figure 2).

The evaluation of BCL of the whole internal dataset will be initially conducted by two spine surgeons (Reader 1: PD with 15 years of experience and Reader 2: JL with 29 years of experience) independently. The inconsistent assessments will be reviewed and adjudicated by a third spine specialist (LZ with 31 years of experience), and the final results will serve as the reference standard. The external dataset will be checked by two spine clinicians (Reader 3: TW with 5 years of experience and Reader 4: QW with 8 years of experience) independently, and inconsistent assessments will also be reviewed and adjudicated by the same spine specialist LZ. Additionally, Reader 3 and Reader 4 labeled the internal test set independently, for further evaluation of interobserver reliability and served as the control group to compare with the DL model. All selected readers are blinded to patients’ demographics, clinical baseline data, and surgical procedures, without any prior information except postoperative images.

### 2.7 Preparation and development of DL model

All algorithms will be conducted in Python 3.8.0 running on the Ubuntu 18.04 operating system, based on the hardware including Intel (R) Xeon (R) CPU E5-2620 V4, Titan V 12G GPU.

The schema of the expected neural network pipeline is shown in Figure 1A. The DL system consists of two main steps: (1) Preprocessing, including resampling, scale adjustment, vertebral segmentation, and bounding box generation; (2) Classification. Our team has once designed an interactive U-net neural network architecture to perform the CT vertebral segmentation task, and the vertebral segmentation Dice coefficient reached 96.8% ± 1.2% (24). The approach begins with an interactive initialization locator module, which allows surgeon manually determined the target vertebrae, followed by automatically segmentation. The interactive design omits the iterative search of freshly fractured vertebrae and avoids the possibility of localization error, especially in patients with multi-level or previous fractures. Further training on MRI data and corresponding U-net neural network architecture optimization will be conducted. Second, a bounding box generation technique (25) was performed to reduce the search area for CNN classifier, and to reserve sufficient information beyond the vertebrae of the initial image such as paravertebral vessels. The bounding box starts with contour detection of segmentation mask (in both axial and sagittal images) and cropped out of the original image (25).

**Figure 3:**
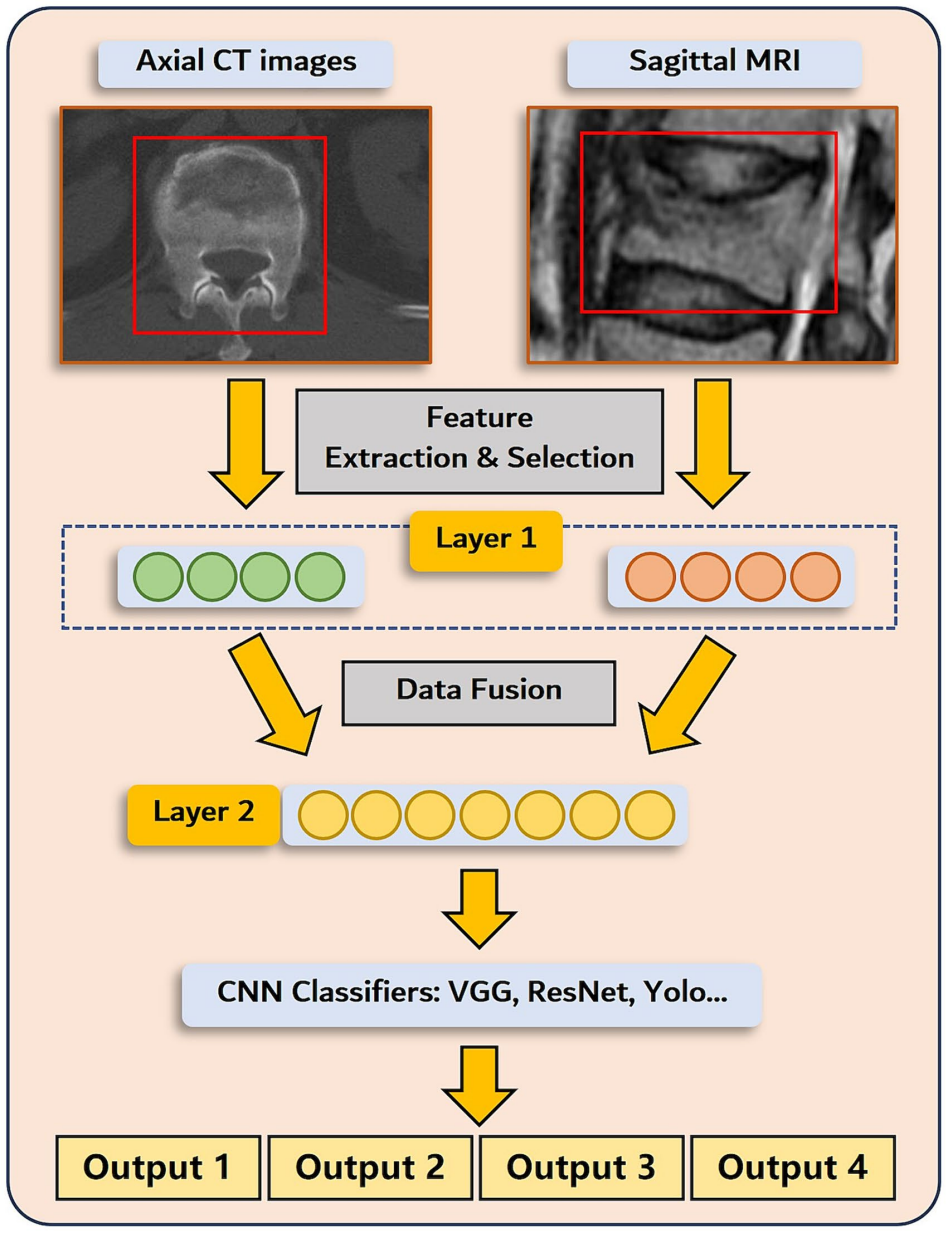
Architecture of the deep learning prediction model.

Next, intermediate multimodal fusion CNN-based classifiers will be designed to integrate the information of axial CT image and sagittal MRI (26). The framework of the applied architecture is shown in Figure 3. The approach begins with transforming input data (preprocessed CT and MR images) into higher-level representations by elaborate feature extraction and selection. A shared representation layer is then fused by the representations from different sources. Finally, several CNN-based classifiers, such as VGG, ResNet, Yolo, etc., were used to perform final multi-task predictions.

### 2.8 Outcomes

The primary outcome measures will be the main exactitude performance of DL model for binary predicting (0/1) different subtypes of BCL, including sensitivity, specificity, and the area under the receiver operating characteristic curve (AUROC), in both internal and external test sets. Secondary outcomes include other exactitude performance such as accuracy, precision, and F1 score. Additionally, the reliability of the model will be evaluated by calculating its consistency with reference standards and comparing it with that of clinician prediction.

### 2.9 Sample size

The data for DL in this study will be a matched series of spinal axial CT and sagittal MRI so that each participant represents one sample. According to a sample size determination study based on DL models and 3D images, the receiver operating characteristic (ROC) curve of classification accuracy became stable when the training sample size increased to 200, and reached a high 98% classification accuracy at a training sample size of 1,000 per class (27). In the present study, binary predictions are warranted for each subgroup of BCL, so at least 250 participants in the internal dataset, containing 200 (80%) for the training set, as the minimal sample size will be acceptable for accurately performing the task. However, more eligible patients up to 1,250 participants, containing 1,000 (80%) for the training set, will be enrolled to achieve a higher accuracy.

### 2.10 Statistical analysis

All data will be analyzed using *Python* 3.8.0. The confusion matrix of DL model and readers in detecting Type I–IV of BCL will be used to evaluate the predicting performance. Quantitative evaluation metrics containing accuracy, sensitivity, specificity, precision, and F1 score, will be calculated based on the confusion matrices. Meanwhile, the ROC curve for binary classification will be plotted and AUROC will be calculated. Linearly weighted Cohen’s kappa coefficient between Reader 3, Reader 4, and the DL model with the reference standard will be used to assess the interobserver and model’s reliability. Statistical significance is set at *p* < 0.05.

### 2.11 Dissemination

The findings of the study will be published in peer-reviewed journals, and national or international conferences. All data related to this study will be kept until 5 years of publication, and available from the corresponding author on reasonable request.

## 3 Discussion

This study aims to develop and validate a DL-based multimodal image analysis model capable of predicting subtypes of BCL using preoperative spine CT and MRI scans of patients with OVCF. The model holds an imperative clinical significance. By preoperatively identifying patients at high risk for each subtype of BCL, clinicians can formulate more targeted treatment strategies to minimize the incidence of BCL thus improve clinical outcomes. Moreover, the model will promote information sharing and decision-making between clinicians and patients efficiently, thereby increasing the overall quality of healthcare services.

A primary concern of spine surgeons during PKP is reducing the occurrence of BCL. Despite rigorous efforts, the rate of BCL remains alarmingly high, even up to 80% (9, 10). This is particularly disturbing as certain types of leakage can cause severe complications, even mortality. Notably, leakage remains an unavoidable risk even if the operator is an experienced specialist. Currently, intraoperative C-arm fluoroscopy is still the most predominant approach for monitoring BCL, yet its image quality is even inferior to that of plain radiographic images (23). This hampers the early and accurate detection of BCL, particularly leakage into the spinal canal, during the intervention. Consequently, developing an effective and reliable preoperative BCL prediction model to facilitate precise individualized risk prediction in patients with OVCF is crucial. To date, only a few studies attempted to predict BCL using several traditional ML models (16–18). Li et al. used six ML models to predict BCL by incorporating clinical baseline characteristics and surgery-related variables, and the AUROC for these models was 0.633–0.898 (16). Further, Hu et al. incorporated radiographic variables from 425 patients to predict BCL using five ML models and revealed that the XGBoost demonstrated the highest AUROC of 0.8819 (17). However, these traditional ML models had inherent limitations, such as depending on manually extracted features and a lack of subtyping for BCL, which restricted their generalization. Recently, an initial attempt has been made to predict BCL using a DL model, but it still relied on manual image feature extraction (17). In addition, the predictive performance of this DL model was inferior than that of the XGBoost model. This indicates that while DL has notable advantages in several domains like image, language, and audio processing, tree-based ML methods have achieved robust predictions more easily than DL for small-scale data (17). However, with the rapid advancements in artificial intelligence technology, it is currently attractive and of great potentiality in developing more accurate and concise DL models by large datasets for predicting BCL.

Notably, different types of BCL result in various secondary complications. However, there is a lack of studies elaborating subtype analysis for BCL, which is one of the chief culprits resulting in the gaps between existing prediction models and real clinical applications. Drawing on the work of Bermejo et al. (9), we propose a modified BCL classification system (types I–IV) based on the location of extra-vertebral bone cement. In Bermejo’s system, basivertebral vein leakage is bracketed in Type S together with segmental vein leakage, and spinal canal leakage is bracketed in Type C together with leakage through cortical defect to elsewhere. However, from a prognosis point of view, basivertebral vein leakage may delay neurological complications despite it is generally safer than through cortical defect. Furthermore, paravertebral vein leakage (excluding basivertebral vein leakage), has been proven as an independent risk factor for pulmonary cement embolism. Therefore, it is reasonable to divide basivertebral vein leakage into spinal canal leakage, as a serious situation to avoid as much as possible. Similarly, Shi et al. developed a nomogram to predict intra-spinal canal leakage, a type same as Type IV in this study, which combined leakage through basivertebral vein with cortical defect (24). Leaking into the spinal canal is among the most severe types of leakage, potentially causing subsequent nerve compression and even disability (28). Consequently, a crucial goal of this modified classification system is to maximize the recall of the DL model on the premise of the overall accuracy, thereby minimizing the risk of mispredictions for spinal canal leakage. This modified classification system is expected to make more accurate preoperative predictions for BCL, providing further targeted clinical guidance and satisfied outcomes.

Overall, our study presents several significant advantages over previous predictive models. First, our model directly uses preoperative CT and MRI images as inputs. Previous studies have emphasized the clinical significance of radiology-related variables such as cortical destruction, intervertebral cleft, bone mineral density, fracture severity, fracture type, and basivertebral foramen sign in BCL prediction (9, 17, 29–31). In particular, Hu et al. determined only preoperative imaging parameters and intraoperative cement volume as independent risk factors for BCL after analyzing over 20 potential risk factors, including baseline characteristics, medical history-, surgery-, and radiology-related variables (17). Hence, a purely image-based DL prediction model is both adequate and reasonable. The DL model adaptively learns feature information from preoperative images without manual intervention, which enables seamless integration into current clinical workflows (32). Second, BCL is an immediate intraoperative complication without being affected by various confounding factors during long-term follow-up, improving the credibility and interpretability of our predictive model. Third, our model not only predicts the occurrence of BCL during PKP, but also enables further subtypes of leakage. Such predictive results are instrumental for clinicians in developing more thorough and personalized treatment plans. Next, our models will be trained on relatively large sample size datasets and validated with prospective, multi-center datasets, which not only improves the performance of the model, but also improves its generalization and applicability across different healthcare settings. Moreover, we will compare the prediction capability of our model with that of clinicians to test its reliability to further evaluate the performance of the model, which will provide a more comprehensive and objective evaluation of the model’s performance.

This study has several limitations. First, the training set for the model is derived from a single-center retrospective dataset, which may introduce selection and subjective bias. Future research should use larger, prospective, and multicenter datasets to train and validate the model, thereby improving its reliability and generalizability. Second, our study solely used preoperative medical imaging data as input, omitting surgery-related variables such as surgical access, puncture pin location, and cement volume, which have been proven to be associated with BCL (9, 17, 29, 31, 33, 34). However, our model is designed to serve as a preoperative prediction tool, to conduct straightforwardly and easily personalized preoperative risk assessment and surgical planning. Finally, as the gold standard for BCL assessment remains postoperative CT examination; thus, not all patients in this study underwent postoperative CT, which may somehow result in inaccuracy of the assessment of BCL.

In conclusion, we will develop a multimodal image analysis model based on DL algorithms for predicting intraoperative BCL in PKP. To the best of our knowledge, it will be the first study to directly utilize preoperative CT and MRI images for BCL subtype prediction. Although there are numerous challenges and unresolved issues in the successful development and application of this model, its potential is highly promising. This approach facilitates individualized and intelligent preoperative assessments for patients with OVCF, providing a crucial basis for surgical decision-making. In particular, the model can be extended and applied in remote areas where medical resources are relatively scarce, so that more patients can benefit from quality perioperative evaluation and management strategies. We will continue to explore the feasibility and limitations of this model in practical clinical applications in future studies, to further improve its accuracy and utility.
